# Bioactive Substances and Biological Functions in *Malus hupehensis*: A Review

**DOI:** 10.3390/molecules28020658

**Published:** 2023-01-09

**Authors:** Pengcheng Li, Jiaqi Tan, Mi Xiao, Xu Cai, Hongkun Xue, Hansong Yu

**Affiliations:** 1 College of Food Science and Engineering, Jilin Agricultural University, Changchun 130118, China; 2 College of Traditional Chinese Medicine, Hebei University, No. 342 Yuhua East Road, Lianchi District, Baoding 071002, China; 3 Pharmaceutical Preparation Section, Union Jiangbei Hospital, Huazhong University of Science and Technology, Wuhan Caidian People’s Hospital, Wuhan 430114, China; 4 Key Laboratory of Particle & Radiation Imaging, Ministry of Education, Department of Engineering Physics, Tsinghua University, Beijing 100084, China

**Keywords:** *Malus hupehensis*, bioactive substances, biological activities

## Abstract

*Malus hupehensis* (MH), as a natural resource, contains various active ingredients such as polyphenols, polysaccharides, proteins, amino acids, volatile substances, and other components. Increasingly, studies have indicated that MH showed a variety of biological activities, including antioxidant, hypoglycemic, hypolipidemic, anti-cancer, anti-inflammatory activities, and other activities. Hence, MH has attracted wide interest because of its high medical and nutritional value. It is necessary to review the active components and biological activities of MH. This paper systematically reviewed the chemical substances, biological activities, and potential problems of MH to further promote the related research of MH and provide an important reference for its application and development in medicine and food.

## 1. Introduction

*Malus hupehensis* (MH) belongs to an important tree or small tree of the genus *Malus* in Rosaceae, and it is widely planted in Hubei, Jiangxi, Shandong, and southern Sichuan of China [1,2]. MH is also called Chinese Begonia or Tea Begonia. Its fruit is spherical with a diameter of 8–12 cm, and its leaves range in length from 5 cm to 10 cm and width from 2.5 cm to 6 cm [3,4]. In addition, MH can also be used as an ornamental plant. MH contains rich chemical active ingredients in branches, leaves, fruits, roots, and other tissues, including polyphenols, flavonoids, proteins, polysaccharides, vitamins, and trace elements [5]. Among them, the research on phenolic components of MH have received extensive attention. It has been reported that phloridzin, phloretin, quercetin, chlorogenic acid, anthocyanins, and proanthocyanidin could be detected in the leaves and fruits of MH [6,7]. Therefore, MH shows a variety of active functions such as antioxidant, hypolipidemic, anti-diabetes, anti-inflammatory, etc. Currently, the research of MH has attracted more and more attention from food and pharmaceutical products.

At present, MH has many varieties in China, such as *Malus* halliana Koehne, *Malus honanensis* Rehder, *Malus micromalus* Makino, *Malus sieboldii* (Regel) Rehder, and *Malus yunnanensis* (Franch.) C. K. Schneid, etc. The tender leaves of MH are often used to make tea in Hubei province, and it is commonly known as “Haitang Tea”, “Sanpi Pass”, “Pingyi Tea”, “Qiuzi Tea”, “Huahei Tea”, etc. [8]. In addition, MH is also widely used as a cool drink in summer. A large number of studies have shown that long-term consumption of MH could help improve blood glucose and blood lipid [9,10], which is a patent of Hubei Province (ZL 200710053716.8) [11].

At present, there is no systematic review on the types of bioactive substances in MH and molecular mechanisms of theirs biological activities. Hence, the nutritional and phytochemical properties of MH were reviewed to support its potential industrial applications, and then the nutritional characteristics of MH were reviewed to support its application prospect as an active ingredient in functional food. In addition, we also put forward the problems that need to be solved in the future research of MH to broaden the application of MH in food and medicine, and promote the further utilization and development of natural resources in Hubei province.

## 2. Compounds in MH

Increasing reports have separated and identified a variety of beneficial bioactive compounds in MH, including polyphenols, flavonoids, pigments, peptides, amino acids, polysaccharides, and volatile compounds. Next, we systematically review the bioactive compounds in MH (Table 1).

### 2.1. Phenolic Compounds

Polyphenol (MH16) is a kind of organic compound widely existing in nature, which is directly connected by hydroxyl and aromatic nucleus (benzene ring or fused benzene ring), and polyphenol (MH16) usually has good biological activities. MH is rich in polyphenol (MH16) in fruits, leaves, roots, and other tissues. The content and composition of total phenols in different regions and tissues are different. The 3-hydroxy phloridzin (MH15), phloretin (MH2), retinoin (MH5), and kaempferol 3-O-β-D-glucoside (MH4) were isolated from the ethyl acetate extract of common green tea-leaves of MH, by the combination of offline two-dimensional high-speed counter-current chromatography (2D HSCCC) strategy and circular elution mode [12]. Liu et al. (2018) quickly screened and identified 32 antioxidants (10 dihydrochalcones, 2 flavanones, 9 flavonols, 4 flavonoids, and 7 phenolic acids) from leaves of MH by combining offline two-dimensional high performance liquid chromatography, ultraviolet and tandem mass spectrometry detection techniques with 1,1′-diphenyl-2-pyridinylmethylhydrazinyl assay [22]. Guo et al. (2018) used thin layer chromatography to identify MH, *Malus toringoides* (MT), and *Malus hupehensis* var. Pinyiensis (MHVP). The content of total flavonoids in MH, MT, and MHVP was measured via UV spectrophotometry, and HPLC was employed to the content of total rutin. The results show that the content of total flavonoids in MH, MT, and MHVP were 19.65–20.97 mg/g, 10.71–12.13 mg/g, and 19.65–20.97 mg/g, respectively. The content of phlorizin (MH1) in MH, MT, and MHVP were 15.51 mg/g, 14.66 mg/g, and 2.05 mg/g, respectively. Compared with MH and MT, MHVP has the highest flavonoid content [23].

Cai et al. (2021) prepared the two solvent systems consisted of n-hexane-ethyl acetate-acetonitrile-water (5:3:5:7, *v*/*v*) and n-hexane-ethyl acetate methanol-water (1:2:1:2, *v*/*v*). High-efficiency enrichment and separation methods were established by liquid–liquid extraction and HPLC. It was found that the two new compounds, namely, 6″-O-coumaroyl-2′-O-glucopyranosylphloretin (MH22) and 3‴-methoxy-6″-O-feruloy-2′-glucopyranosylphloretin (MH23), were obtained from MH [16]. Response surface analysis (RSM) is an optimization method, which takes the response of the system (such as the extraction rate in chemistry) as a function of one or more factors (such as the concentration of extractant, acidity, etc.), and uses graphical techniques to display this functional relationship, so that we can use intuitive observation to select the optimal conditions in the test design [24,25]. RRSM was used to study the extraction of green and efficient flavonoids based on deep eutectic solvent (DES). DES refers to a two component or three component low eutectic mixture composed of hydrogen bond receptors (such as quaternary ammonium salt) and hydrogen bond donors (such as amide, carboxylic acid, polyol and other compounds) with a certain stoichiometric ratio. DES is a new almost non-toxic green reagent. Its concept was first proposed by Abbott [26], and its properties are similar to ionic liquids. However, compared with ionic liquids, DES is simpler, faster, cheaper, and less toxic. It has developed rapidly in the last five years and has been used more and more widely [27]. A high-speed counter-current chromatography solvent system based on DES was developed for the first time to separate high-purity compounds from MH. The results show that five flavonoids were effectively separated from MH, including 1-2, 6″-O-coumaroyl-2′-O-glucopyranosylphloretin, 3‴-methoxy-6″-O-feruloy-2′-O-glucopyranosylphloretin (MH23), and three known compounds (phlorizin (MH1), avicularin (MH3), and sieboldin) [17]. The content of polyphenol (MH16) in MH could affect the change in plant color. The leaves of MH could change from green to reddish brown during the development process. Red leaves are an attractive feature of the *Malus* family, including crabapple (*Malus* spp.). The relative accumulation of these compounds in *Malus* leaves is related to the balance of gene expression. Overexpression of McDFR or silencing of McFLS could increase the production of anthocyanins. The relative activities of McDFR and McFLS in MH are the basis for its production of polyphenol (MH16) compounds. Hence, the polyphenol (MH16) content in leaves and fruits of MH can be changed by regulating these enzymes [28].

### 2.2. Polysaccharides

Polysaccharides are high-molecular weight carbohydrates formed by more than 10 monosaccharides through glycosidic bonds, often with a variety of biological activities. Plants are one of the most important sources of natural polysaccharides. MH contains a large number of polysaccharides with biological activities. Li et al. (2021) extracted and separated polysaccharides from the leaves of MH by using ultrasonic-assisted aqueous two-phase method, and characterized the structure of the polysaccharides in the lower phase. The results show that the monosaccharides of MH consisted of Man (3.22%), GlcA (1.85%), Rha (4.25%), GalA (5.08%), Glc (6.69%), Gal (38.46%), Xyl (4.68%), Ara (30.90%), and Fuc (4.87%). The results of Congo red test and circular dichroism show that the polysaccharide of MH had asymmetric and triple helix structure [29]. It is worth noting that there are relatively few studies on the polysaccharides of MH. In addition, the research on the structure, physicochemical properties, and biological activities of MH polysaccharides is not enough. Therefore, it is hoped that researchers in relevant fields can further study MH polysaccharides and promote theirs utilization and development.

### 2.3. Volatile Components

Volatile substances are mainly organic compounds, including ketones, acids, esters, alcohols, and other substances, which gives MH a special aroma and unique flavor. A large number of studies have confirmed that the volatile substances content of MH (esters, ketones, alcohols, and acids) was relatively low, whereas a low content of volatile substances has strong biological activities. At present, the volatile substances in MH as functional factors are added to functional food and drugs to maximize the development and utilization of MH [30].

### 2.4. Vitamin

Vitamins are a kind of organic compounds necessary for maintaining health. This kind of substance is neither the raw material of body tissue nor the source of energy in the body. However, vitamins are a kind of regulatory substances and play an important role in human growth, metabolism, and development. Plants are one of the most important sources of vitamins. Mounting evidence has indicated that MH contains various vitamins (C, B1, B2, B6, and nicotinic acid) in leaves, roots and fruits [31].

### 2.5. Other Ingredients

Increasing studies have confirmed that the active substances in MH also include lipids, pigments, trace elements, protein, dietary fiber, etc. [32,33,34,35,36]. At present, there are relatively few studies on the above compounds, whereas these substances also showed high biological activities. Therefore, these substances have good prospects for development and utilization. A previous study has reported that dietary fiber has become the seventh largest nutrient, which could regulate blood sugar and blood lipids, moisten the intestines and relieve constipation. With the continuous progress of science and technology, it is still necessary to further explore the physiological activities and molecular mechanisms of the above substances in the future to maximize the use of MH.

## 3. Biological Activities of MH

**Figure 1:**
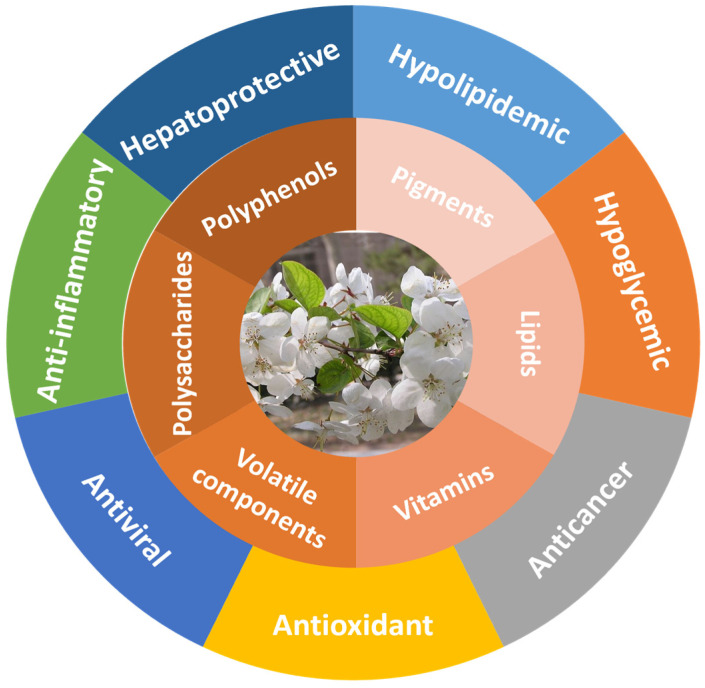
Bioactive substances and physiological activities of MH.

MH is rich in many active ingredients, which lays a material foundation for its rich biological activities. Hence, MH shows a variety of biological activities, including antioxidant, anticancer, hypoglycemic, hypolipidemic, hepatoprotective, and other activities. At present, more and more studies have indicated that MH could be developed as functional food and medicines to prevent and treat various chronic diseases. Figure 1 summarizes the biological activities of MH.

### 3.1. Antioxidant Activity

In antioxidant research, DPPH, ABTS, OH, O^2-^ free radicals scavenging capacity and other methods are usually used to evaluate the antioxidant activity of target substances in vitro, and cell models can also be further established to determine theirs antioxidant activity. In addition, animal models are often used to evaluate the antioxidant activity of target substances in vivo.

Liu et al. (2019) studied the antioxidant activity of MH extracts (MHEs) in vitro, and conducted the antioxidant experiments by using the aging model of rats in vivo. The results show that the MHEs had high antioxidant activity, and the IC_50_ values of DPPH, ABTS, O^2−^, and tyrosinase inhibition of MHEs were 19.00 μg/mL and 303.94 μg/mL, 3.71 mg/mL, and 1.16 mg/mL, respectively. Moreover, MHEs could significantly increase the activities of antioxidant enzymes in serum and tissue homogenate of rats in a dose-dependent manner [37]. Liu et al. (2018) prepared 32 antioxidants from MH, including 10 dihydrochalcones, 2 flavanones, 9 flavonols, 4 flavonoids, and 7 phenolic acids, and evaluated theirs antioxidant activity. It was found that 32 antioxidants could significantly eliminate excessive free radicals [22]. Hu et al. (2018) isolated twenty-three compounds from the leaves of MH, including twenty-one known polyphenols and two new compounds (4-chromanone glycoside, 5-O-β-d-glucopyranoside-4-chromanone (MH24)), and the SC_50_ values of the compound with the strongest antioxidation were 2.73 μg/mL. By analyzing the HPLC fingerprints of leaf and fruit extracts, it was found that the components in the two kinds of extracts were almost the same except for phlorizin (MH1) content [18]. Wang et al. (2013) extracted and separated three new flavonoid glycosides and twelve known flavonoid compounds from the leaves of MH. The antioxidant activity and protective effect of these compounds against doxorubicin-induced cardiomyopathy in H9c2 cells were also studied. Among them, quercetin (MH6) was the most active free radical scavenger, and the EC_50_ values of DPPH and ABTS were 3.2 μM and 17.8 μM, respectively. Three new compounds also had strong protective effect against cell death, and EC_50_ values were 8.3, 5.2 and 7.6 μM, respectively [13]. Jin et al. (2014) evaluated the antioxidant and anti-inflammatory effects of ethanol extracts from MH, Ophiorrhiza cantonensis, and Psychotria rubra. The three extracts and ascorbic acid had significant scavenging capacity for DPPH free radicals. The three extracts could significantly inhibit the levels of ROS in RAW264.7 cells induced by LPS in a dose-dependent manner. In addition, the three extracts could regulate the transcription factor (NF-κB) and activator protein-1 (AP-1), and down-regulate inducible NO synthase (iNOS) to inhibit LPS-induced NO formation. The three extracts had good antioxidant activity in vivo and in vitro.

However, their clinical application is limited due to their poor solubility, fast metabolism, and low bioavailability [38]. Liposomes can be used as a new type of transdermal drug delivery system to effectively deliver active ingredients. Liposomes had better skin penetration potential, retention capacity, and stability, and the safety was also reliable. Therefore, liposomes could greatly improve the utilization value of MH [6]. Li et al. (2019) evaluated the antioxidant activity of the extracts from tea in Wushan. The results showed that the extracts contained quercitrin (MH25), isoquercitrin (MH26), chlorogenic acid (MH27), neosperidin Dihydrochalcone (MH28) by using HPLC analysis. The extracts had good scavenging activity against DPPH, ABTS, and OH free radicals in a dose-dependent manner. In addition, the extracts could significantly down regulate inflammatory related factors such as NF-κB and COX-2 [19]. Oxidized free radicals could cause inflammation of cells, and inflammatory factors could cause cells to produce more oxidized free radicals, forming a vicious circle. So, in many cases, anti-inflammation and anti-oxidation are inseparable. Antioxidant effect can alleviate inflammation to a certain extent. Nithiya et al. (2016) determined the antioxidant activity of phloretin (MH2) in vitro by scavenging capacity of DPPH and OH free radicals, metal chelating activity, and total antioxidant capacity. The IC_50_ values of phlorizin (MH1) on DPPH, OH free radicals’ scavenging capacity, metal chelating activity, and total antioxidant capacity were 48.56 μg/mL, 44.16 μg/mL, 44.59 μg/mL, and 44.83 μg/mL, respectively [39]. Phloretin (MH2) had good inhibitory effects on hydroxyl radicals and lipid peroxidation.

In addition, phloretin (MH2) could inhibit activities of Matrix metallopeptidase 1 (MMP-1), elastase and enzymes to degrade connective tissue, which played an important role in photoaging. Floretin could enhance the absorption of vitamin C and ferulic acid in the skin to increase the concentration of L-ascorbic acid and tocopherol, which played a synergistic role with sunscreen in the light protection of human skin [40]. Hu et al. (2021) evaluated the effects of phloretin (MH2) on growth performance, Nrf2, GSH related enzymes, antioxidant properties, serum biochemical parameters, and HSP70 of heat stressed broilers. The results show that phloretin (MH2) intervention could increase the levels of GSH, CAT, and total antioxidant capacity (T-AOC), and it decreased the feed-to-weight ratio (FGR) and serum malondialdehyde (MDA) levels in broilers, indicating that phloretin (MH2) can enhance the antioxidant properties and growth performance of heat-stressed broilers by regulating the expression of enzymes [41]. H_2_O_2_ could increase the levels of oxidative stress in cell tissues, and phyretin (10 μM) could improve the levels of ROS induced by 500 μM H_2_O_2_. Phyretin could improve the activity of SOD, restore mitochondrial membrane potential, and alleviate cell apoptosis and necrosis. In addition, phloretin (MH2) could also regulate the mRNA expression of cyclin D1, HO-1, and Nrf2. Furthermore, phloretin (MH2) could also alleviate the G1 phase arrest trend of cell proliferation, and improve the inhibition levels of Nrf2, p-AMPK, and p-LKB1. Therefore, phloretin (MH2) could play an antioxidant role in C2C12 cells through the LKB1/AMPK/Nrf2/HO-1 pathway [42].

**Figure 2:**
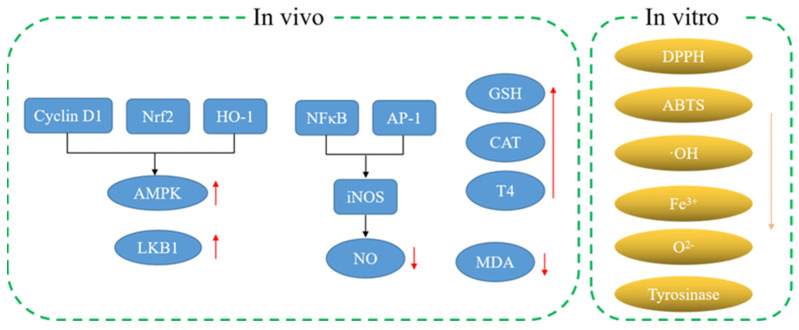
Antioxidant mechanism of MH.

A previous report has found that MH contained melatonin (MH34), and melatonin (MH34) was considered as a specific neurotransmitter in vertebrates. The results show that melatonin (MH34) could be used as a plant growth regulator on the one hand. In addition, melatonin (MH34) could also be used as an antioxidant and growth promoter. Furthermore, melatonin (MH34) could stabilize the redox state of cells and protect tissues from oxidative stress [20]. Based on the above analysis, MH has good antioxidant activity, which is mainly due to the rich active substances in its roots, stems and leaves. For example, phenolic compounds with more unsaturated bonds showed excellent antioxidant activity. Therefore, MH can be used as a natural source of antioxidants and a promising material in the field of nutrition. Moderate intake of MH can improve antioxidant capacity and promote health (Figure 2).

### 3.2. Anticancer Activity

Compared with normal cells, cancer cells contain three characteristics, such as unlimited proliferation, transformation, and easy metastasis. Cancer cells can proliferate indefinitely, destroy normal cell tissues, and even metastasize to other parts through the circulatory system or lymphatic system. Cancer is one of the major diseases that seriously affect human health and threaten human life. With the rapid development of science and technology, cancer diagnosis technology is becoming more and more advanced. Many cancers can be diagnosed accurately, whereas the current treatment technology is still helpless for many cancers. At present, treatment includes surgery, chemotherapy, radiotherapy, endocrine therapy, targeted therapy, immunotherapy, interventional therapy, and so on, among which surgery, chemotherapy and radiotherapy are the main means. However, surgery, radiotherapy or long-term use of chemical drugs will lead to the decline of body immunity, and cause great trauma to the body and mind of patients. MH, as a highly effective, non-toxic and side effect-free natural anti-tumor active substance, can be used to inhibit the growth of tumor cells and accelerate apoptosis, which is helpful to prevent and treat various cancers. MH, as a traditional Chinese drink, is favored for its unique flavor and medicinal functions.

The extracts from MH could increase HepG2 cells apoptosis. In addition, MH could up-regulate the levels of caspase-3, caspase-7, caspase-8, caspase-9, p21, p53 and Bax proteins, and down-regulate the levels of Bcl-2 protein to achieve inhibition of HepG2 cell growth [19]. Seven dihydrochalcones were isolated from MH and identified their chemical structures by using UV, FT–IR, ESI-MS, ^1^H-NMR, and ^13^C-NMR. The results show that two new compounds (6″-O-coumaroyl-4′-O-glucopyranosylphloretin (MH36) and 3‴-methoxy-6″-O-feruloy-4′-O-glucopyranosyl-phloretin (MH37)) were obtained. These compounds had good inhibitory effects on A549, HepG2, Bel7402, and HT-29 cancer cell lines [21]. Yoshizawa et al. (2007) prepared 42 *Malus* spp. juices and found that most of the juices had a strong inhibitory effect on the proliferation of HL-60 human leukemia cells. The scavenging activity of DPPH free radicals was positively correlated with the anti-tumor activity of HL-60 cells [43].

Phloretin (MH2) could enhance the anti-tumor activity of immune cells, which is beneficial to the proliferation and differentiation of immune cells. The growth of cancer cells could be significantly inhibited by using phloretin (MH2) to treat cancer cells. The mechanism of action was related to the increase cell apoptosis rate, the activation of caspase-9 and caspase-3 proteins, the increased levels of PARP cleavage and Bax, the up-regulation levels of p53 and the reduction in Bcl-2, which led to DNA fragmentation and cell death [44]. Phloretin (MH2) (0.15 mM) could accelerate the apoptosis of melanoma 4A5 cells in B16 mice. Quercetin (MH6) mainly inhibited the activity of protein kinase C in B16 mouse melanoma 4A5 cells and accelerated cell apoptosis. The expression levels of COX-2 protein were related to multiple malignant tumors. Topical application of phloretin (MH2) could reduce the number of skin tumors by 40% and the size of tumors by half. Local application of phloretin (MH2) for 18 weeks could down-regulate the expression levels of COX-2 protein [45].

**Figure 3:**
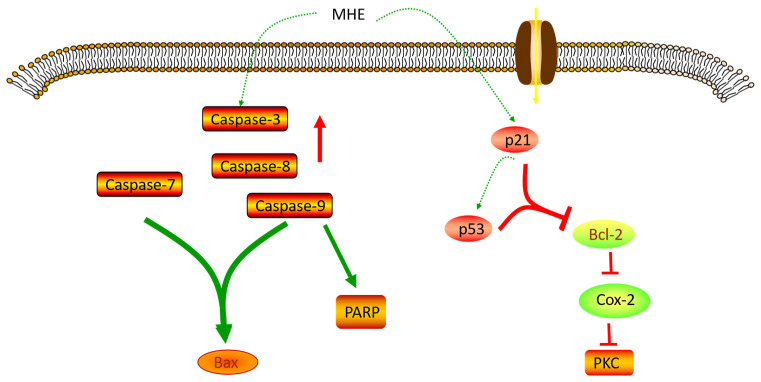
Anticancer mechanism of MH.

The remarkable anticancer activity of MH is attributed to the abundance of various active compounds. However, the molecular mechanisms of the specific biological activities of these compounds still need further study (Figure 3).

### 3.3. Hypoglycemic Activity

Hyperglycemia refers to fasting blood glucose (FBG) or postprandial blood glucose (PGB) exceeding the positive range, which can be manifested as polydipsia, polyuria, overeating, weight loss, and other symptoms. However, most patients with hyperglycemia have no obvious symptoms. Long term hyperglycemia can lead to complications such as blurred vision, limb numbness and pain. With the continuous improvement of living pressure and living standards, the incidence rate of hyperglycemia and diabetes is on the rise and tends to be younger. China has a long tradition of tea culture. Polyphenol (MH16) and other substances contained in tea have certain effects on preventing hyperglycemia or alleviating diabetes.

The leaves of MH are often used as tea for drinking. Three dihydrochalcones and thirteen known compounds were isolated from MH. The hypoglycemic effect of these compounds was evaluated by the inhibitory activity test of α-glucosidase, and it was found that phlorizin (MH1), 3-hydroxyphloridzin, 3-O-coumaroylquinic acid (MH17) and β-hydroxypropiovanillone (MH18) showed significant inhibitory effects on α-glucosidase. These compounds may be the reason for the typoglycemic effect of this herb [15].

Many studies have shown that phlorizin (MH1) has a good effect in the treatment of diabetes [46,47,48]. Increasingly, researchers have been using phlorizin (MH1) when they studied the pathogenesis of diabetes and explored ways to treat diabetes, which made the research on treating diabetes fast and smooth. Wang et al. (2021) studied the effects of phlorizin (MH1) and the main component in MH leaves on blood glucose and glucose-6-phosphatase (G-6-Pase) to provide a scheme for blood glucose stability. The experimental results show that phlorizin (MH1) could promote the glucose uptake of insulin resistant HepG2 cells, improve the glucose tolerance of mice, and enhance the levels of FBG and PBG, and inhibit the expression levels of G-6-Pase protein, which had a positive effect on stabilizing the blood glucose of mice [49].

The development of analytical technology has laid an important foundation for chromatographic separation and activity evaluation of complex compound systems. XI et al. (2021) has established a rapid and efficient screening platform for glucosidase inhibitors, and applied it to screen and evaluate the active components in MH. There are 6 kinds of glucosidase inhibitors, including quercetin (MH6). The establishment and successful application of this platform provide a powerful tool for effective discovery of anti-diabetes active ingredients in complex systems [50]. The intervention of Phlorizin (MH1) could significantly improve the abnormal levels of glutathione, brain-derived neurotrophic factor, MDA, acetylcholinesterase, extracellular signal-regulated kinase (ERK), tyrosine receptor kinase B (TrkB), and cAMP response element binding protein (CREB) in the brain of diabetes rats, which could effectively improve oxidative stress level and depression in diabetes rats [51,52]. Zhang et al. (2021) found that phlorizin (MH1) could reduce the levels of fasting blood glucose in type 2 diabetes rats (T2DM), improve the levels of serum lipid, reduce the damage of pancreatic islet cells and reduce the accumulation of fat in liver cells of HFD mice, suggesting that phlorizin (MH1) shows good therapeutic and preventive effects on hyperglycemia and hyperlipidemia in diabetes rats [53]. Liang et al. (2016) proved that phlorizin (MH1) could reduce the levels of water consumption, body weight, FBG, FINS, HOME-IR, serum leptin and CRP, and increase the levels of serum adiponectin.

**Figure 4:**
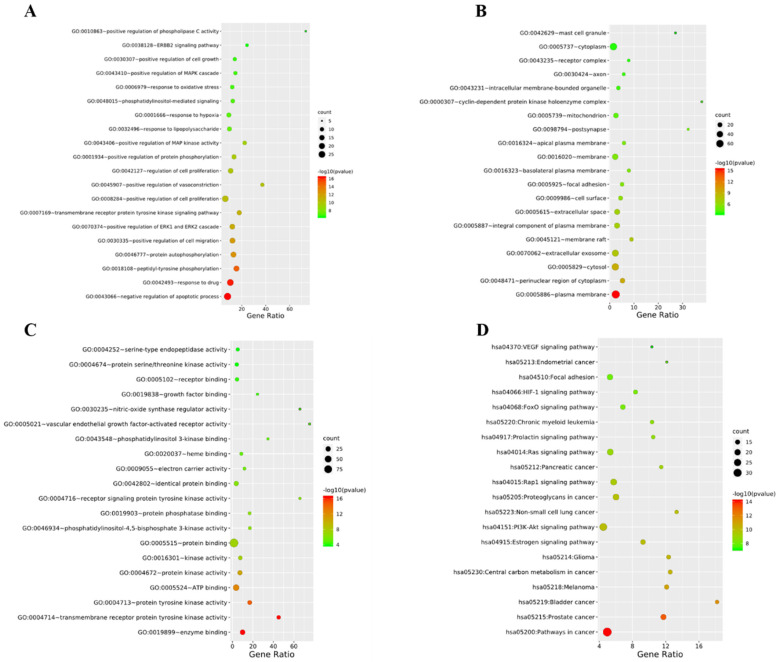
Biological process (**A**), molecular function (**B**), action target (**C**) and involved pathway (**D**) that may be involved in the effect of MH on regulating blood glucose.

In addition, phlorizin (MH1) could improve the oral glucose tolerance test to a certain extent in diabetes mice. Furthermore, phlorizin (MH1) could also reduce the liver index, epididymis, and white adipose tissue index around the kidney, and increase the pancreas index of diabetes mice and the levels of glycerol kinase (GK) and glycogen in the liver, and reduce the activities of PEPCK and G-6-Pase, and effectively improve the symptoms of diabetes [54]. Cai et al. (2013) found that phlorizin (MH1) could significantly reduce the weight gain and the levels of FBG, TG, TC, and AGEs in db/db mice. The treatment of phlorizin (MH1) could change the protein expression levels related to heart injury by regulating heart lipid and energy metabolism to maintain normal myocardial structure and prevent diabetes cardiomyopathy [55]. Phlorizin (MH1) could reduce PBG and FBG, whereas phlorizin (MH1) would not cause hypoglycemic side effects. Treatment of diabetes rats with phlorizin (MH1) could make their insulin sensitivity return to normal, and eliminate or reduce insulin resistance caused by glucose toxicity [56,57,58]. In addition, phlorizin (MH1) could also prevent albuminuria and renal hypertrophy [59]. Sodium glucose symporter (SGLT) is a widely distributed membrane protein responsible for glucose transport in the body. Phlorizin (MH1) could specifically and competitively inhibit the transport of glucose molecules by SGLT-1 and SGLT-2. SGLT-2 was mainly expressed in the kidney, while SGLT-1 was mainly expressed in the intestine and partly in the kidney. SGLT-2 played a major role in glucose reabsorption. About 90% of glucose was reabsorbed through SGLT-2, and only about 10% of glucose was reabsorbed through SGLT-1. Therefore, SGLT-2 inhibitors could block the reabsorption of glucose by proximal convoluted tubules and excrete excess glucose through urine, thus reducing blood sugar [60]. SGLT inhibitors could reduce blood glucose by inhibiting glucose reabsorption of transporter tissues, and eliminate carbohydrates from urine. This method of reducing blood glucose did not involve the use of insulin. Therefore, SGLT inhibitors could be used at any stage of T2DM, and this mechanism was also considered to be one of the most promising treatment methods, which could help diabetes patients improve blood sugar control [61]. The substances with hypoglycemic activity in MH also included quercetin (MH6), farnesin, chrysin (MH8), and catechol. Oral administration of quercetin (MH6) and local application of quercetin (MH6) ointment in diabetes rats could normalize the levels of blood glucose, hydroxyproline, and glucosamine. Local application of quercetin (MH6) ointment was beneficial to the wound recovery of diabetes rats [62]. We predicted the hypoglycemic effect of MH based on online pharmacological research. The SwissTargetPrediction platform was used to screen and predict the composition target, and disease targets were obtained from Genecards, OMIM, DrugBank and TTD databases. The DAVID platform was used to draw PPI, and the Cytoscape 3.8.0 was used to make network diagrams. The obtained data were enriched and analyzed by GO and KEGG (Figure 4). Figure 4A–C show that the main biological processes involved in the regulation of T2DM by MH are negative regulation of apoptotic process, peptidyl tyrosine phosphorylation, protein autophosphorylation positive regulation of cell migration and positive regulation of ERK1 and ERK2. Its molecular functions mainly include enzyme binding, transmembrane receptor protein tyrosine kinase activity, protein tyrosine kinase activity and protein kinase activity. The main targets are plasma membrane, perinuclear region of cytoplasm, cytosol and extracellular exosome. The pathways involved mainly include cancer pathway, estrogen signaling pathway, PI3K Akt signaling pathway, etc., shown in as Figure 4D. Phlorizin (MH1), as a natural SGLT inhibitor, was expected to improve blood glucose control in diabetes patients, but there are still some problems such as low oral bioavailability of phlorizin (MH1). In addition, phlorizin (MH1) showed inhibitory activity on SGLT1 and SGLT2. Other tissues and organs had a certain impact on the absorption and utilization of glucose when SGLT1 was largely inhibited [63]. Hence, we still need to conduct more in-depth research on phlorizin (MH1), and consider whether it is possible to modify phlorizin (MH1) molecules, which leads to phlorizin (MH1) derivatives having higher bioavailability. We can obtain natural candidate drugs for the prevention and treatment of diabetes and bring more benefits to diabetes patients.

### 3.4. Hypolipidemic Activity

Hyperlipidemia refers to the abnormal metabolism or operation of fat, which makes the blood lipid content in human blood exceed the normal range. It is characterized by high cholesterol and/or triglycerides (TG) or low high-density lipoprotein (HDL) in the blood, which is called “dyslipidemia” in modern medicine. Hyperlipidemia is a common and frequently occurring disease, which is also the culprit of cardiovascular and cerebrovascular diseases. In addition, hyperlipidemia can directly damage and accelerate systemic atherosclerosis. Long-term lipid-lowering treatment can reduce the incidence rate and mortality of coronary heart disease, angina pectoris, myocardial infarction, stroke, and diabetes. MHE has been proved to improve lipid metabolism disorder and inflammatory response in obese mice induced by high-fat diet. MHE could reduce the weight and fat accumulation of mice and liver damage. In addition, MHE could reduce the levels of ALT, AST, AKP, TC, TG, LDL-C, and improve the levels of HDL-C. After MHE intervention, the inflammatory cytokines in mice significantly reduced, including TNF-α, IFN-γ, IL-1β, and IL-6. However, MHE could significantly increase the anti-inflammatory cytokines (IL-10 and IL-4). This is mainly attributed to the fact that MHE could up-regulate the levels of LPL, CPT1, CYP7A1, SOD1, SOD2, CAT, GSH1 and GSH-Px in the liver of obese mice. In addition, MHE could inhibit the mRNA expression levels of PPAR and C/EBP, thereby playing an anti-obesity role, relieving dyslipidemia, chronic low-grade inflammation, liver injury and other symptoms [10]. A previous study has confirmed that phlorizin (MH1) could significantly alleviate the hyperlipidemia and oxidative stress state of HFD mice (C57BL/6J), and also enhance the intestinal microbiota homeostasis, effectively improving the obesity and health of HFD mice [64].

**Figure 5:**
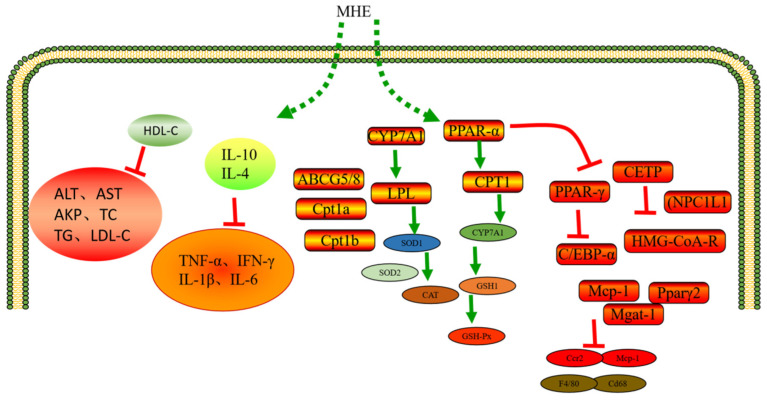
Hypolipidemic mechanism of MH.

Lang et al. (2022) used phlorizin (MH1) to interfere with golden Syrian hamsters. It was found that phlorizin (MH1) could increase the expression levels of CYP7A1, showing significant blood lipid lowering and antioxidant activities [65]. Phlorizin (MH1) showed a very good activity to reduce plasma TC, TG, CETP, and HMG CoA-R levels. In addition, phlorizin (MH1) could down-regulate gene expression levels of NPC1L1 and HMG-CoA-R, whereas phlorizin (MH1) could up-regulate ATP-binding cassette transporters subfamily G members 5/8 (ABCG5/8) gene to increase the excretion of sterols and mediate the endogenous cholesterol metabolic management, which possessed cholesterol-lowering activity [66]. Phlorizin (MH1) has good inhibitory effect to leptin, adipose, TNF-α and IL-6, etc. It is beneficial to prevent diet-induced obesity, liver steatosis, inflammation and fibrosis, and insulin resistance [67]. RT-PCR analysis showed that phloretin (MH2) treatment could enhance the expression levels of adiponectin gene in white adipose tissue, and inhibit the expression levels of proinflammatory genes (Mcp-1, Ccr2) and macrophage markers (F4/80, Cd68). In addition, phloretin (MH2) could reduce expression levels of Mcp-1, Pparγ2, and Mgat-1 genes, increase the expression levels of fatty acid oxidation genes such as Cpt1a and Cpt1b, which effectively relieved obesity and maintained metabolic stability [68]. Quercetin (MH6) and cyanidin-3-glucoside could reduce the concentration of cholesterol in red blood cells and increase the fluidity of cell membrane [69]. Quercetin (MH6) could also reduce the dyslipidemia caused by ethanol, improve the mitochondrial dysfunction caused by ethanol, and increase the activities of glutathione, SOD, GSH-Px. In addition, quercetin (MH6) could reduce ROS production and liver damage [70]. Quercetin (MH6) could also reduce the toxicity of Lindane by regulating the lipid levels, which made the serum TC, TG, LDL, VLDL, and HDL of rats return to normal levels [71]. Jeong et al. (2012) reached a similar conclusion that quercetin (MH6) could significantly reduce the content of MDA in C57BL/KsJ db/db mice, and increase the activities of SOD, CAT, and GSH-Px, which had a good effect on improving the hyperglycemia, dyslipidemia, and antioxidant status of T2DM [72] (Figure 5).

### 3.5. Hepatoprotective Activity

The liver is an organ with metabolic function as its main function, which plays an important role in the body, such as antioxidation, storage of liver glycogen, synthesis of secreted proteins, etc. However, liver disease has always been a common disease among Chinese residents, making China a large country with a high incidence of liver disease.

**Figure 6:**
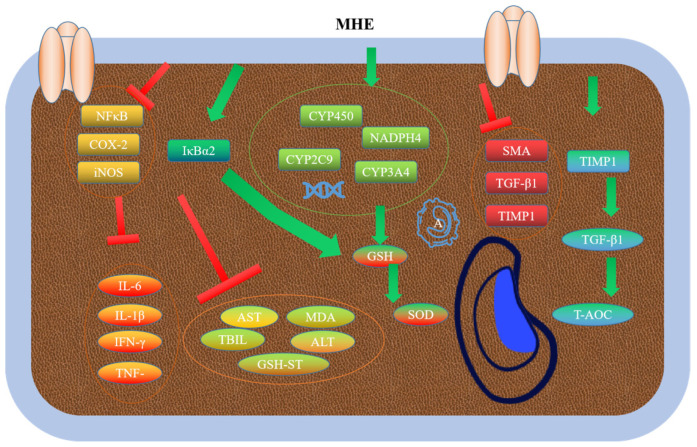
Hepatoprotective mechanism of MH.

MHE could significantly reduce the levels of ALT, AST, TBIL and MDA, and improve the activities of GSH-Px, SOD, and GSH. In addition, MHE could reduce pathological tissue damage. Furthermore, MHE could inhibit the expression levels of CYP450, CYP2C9, CYP3A4, and NADPH4 proteins [73]. MHE could well alleviate the liver fibrosis induced by carbon tetrachloride (CCl4). MHE could increase the levels of AST and ALT in serum, and the activities of SOD, CAT, and GSH-Px. In addition, MHE could reduce the liver coefficient, pathological changes, and the levels of MDA, IL-6, IL-1β, IFN-γ, and TNF-α in CCl4-treated mice. High expression levels of NF-κB, COX-2, and iNOS could promote liver inflammation in mice, whereas MHE could inhibit the expression levels of IκBα to alleviate inflammation [74]. Phlorizin (MH1), phloretin (MH2), and quercetin (MH6) could reduce the ALT, AST γ-GT, and ALP in serum, and improve the pathological changes in liver tissue. Moreover, phloretin (MH2) could also enhance adipocyte differentiation and adiponectin expression in 3T3-L1 cells, and inhibit the expression levels of endothelial adhesion molecules [75]. A previous study has reported that phlorizin (MH1) could significantly reduce liver tissue hydroxyproline and TGF-β1, and increase T-AOC of liver tissue. Additionally, phlorizin (MH1) could down-regulate the expression levels of α-SMA, TGF-β, and TIMP1 proteins, whereas phlorizin (MH1) could up-regulate the expression levels of MMP1 protein. By regulating the expression of these proteins, phlorizin (MH1) could enhance the antioxidant capacity of liver tissue, reduce the level of lipid peroxidation, protect liver cell membrane from damage, and reduce liver fibrosis [76] (Figure 6).

### 3.6. Other Biological Activities

In addition to the above activities, MH also shows various biological activities including anti-inflammatory, neuroprotective, antiviral, bone metabolism promotion, and also other activities. Phloretin (MH2) could inhibit the levels of IL-6, IL-8 and TNF-α. Moreover, MH could stimulate HaCaT cells to release IL-1β and IL-12 to alleviate inflammation [45]. Phlorizin (MH1) improved memory impairment induced by insulin resistance [77]. LPS could reduce the levels of antioxidants (SOD and GSH), BDNF, and AChE in hippocampus and cerebral cortex of animals. However, phlorizin (MH1) could alleviate the changes in these indicators, reduce LPS-induced neuroinflammation and memory disorders, and play a neuroprotective role after intervention [52]. The total flavones of MH could significantly reduce the content of calcium and phosphorus in urine of ovariectomized rats. Moreover, the total flavones could increase the content of serum calcium and estradiol, promote the growth of bone trabecula, increase its thickness and area, and the bone density of ovariectomized rats. The active substances of MH and their biological activities are listed in the article. In the future, MH may also have other bioactive substances and physiological activities, which still need further research and exploration by relevant researchers.

## 4. Conclusions and Future Prospects

The incidence rate of diabetes, cardiovascular disease, immune disease, and other diseases is increasing year by year. These chronic diseases are attracting more and more attention all over the world. The Health Commission has issued various policies or measures to provide better health solutions for everyone. Medicine and food homology food and health care products are widely used in people’s daily life, which provides an important material basis for protecting people’s health. The abundant bioactive substances in plants provide a material basis for their good biological activity. In 2014, China approved MH as a new food raw material, which shows that MH can be eaten completely as food. We summarized the related research of MH, summarized its bioactive substances and bioactivity, and showed that MH contains rich bioactive substances and excellent bioactivity. However, there are still several problems that are worthy of our in-depth study before they can be resolved. First of all, the bioactive substances in MH have not been completely separated and extracted, which requires further development of separation and preparation technology. Secondly, the extraction and application of bioactive substances are mainly concentrated in the laboratory research stage, which cannot be industrialized for mass production and application, and requires close cooperation between researchers and enterprises to experiment with this application. Therefore, we can use biological modification, physical encapsulation or nano delivery technology to improve its bioavailability. In addition, we can also use modern advanced processing technology to produce and process functional food with high added value, which is more conducive to promoting the high value utilization of MH and the development of healthy food.

In conclusion, a large number of studies have laid the foundation for the application of MH in functional food, medicine and cosmetics, but we need to further explore the molecular mechanism of biological active substances to promote the research and application of MH. This review can help readers better understand MH and provide some reference for MH research, application and value development.

## Figures and Tables

**Table 1:** The bioactive substances in MH.

Number	Chemical Compound	Structure	Reference
MH1	Phlorizin	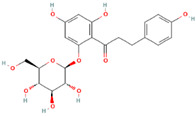	[12]
MH2	Phloretin	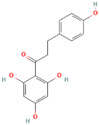	[12]
MH3	Avicularin	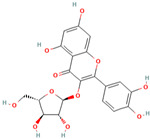	[12]
MH4	Kaempferol-3-O-β-D glucoside	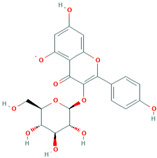	[12]
MH5	Retinoin	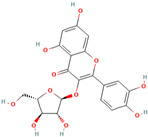	[12]
MH6	Quercetin	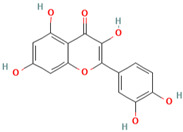	[13]
MH7	Acacetin	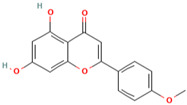	[13]
MH8	Chrysin	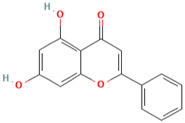	[13]
MH9	5,7-dihydroxychromone-7-O-β-d-glucoside	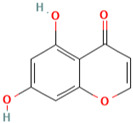	[13]
MH10	Quercetin-3-O-β-d-glucopyranoside	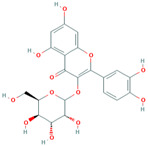	[13]
MH11	Luteolin-5-O-β-d-glucopyranoside	NO	[13]
MH12	Phloretin-2′,4′-di-O-β-d-glucopyranoside	NO	[13]
MH13	Epipinoresinol-4-β-d-glucoside	NO	[13]
MH14	Protocatechuic acid	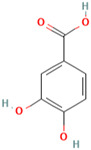	[14]
MH15	3-hydroxyphloridzin	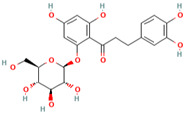	[15]
MH16	Polyphenol	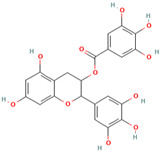	[15]
MH17	3-O-coumaroylquinic acid	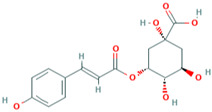	[15]
MH18	β-hydroxypropiovanillone	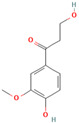	[15]
MH19	Huperolides A	NO	[15]
MH20	Huperolides B	NO	[15]
MH21	Huperolides C	NO	[15]
MH22	6″-O-coumaroyl-2′-O-glucopyranosylphloretin	NO	[16]
MH23	3‴-methoxy-6″-O-feruloy-2′-glucopyranosylphloretin	NO	[17]
MH24	5-O-β-d-glucopyranoside-4-chromanone	NO	[18]
MH25	Quercitrin	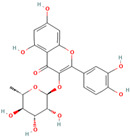	[19]
MH26	Isoquercitrin	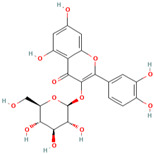	[19]
MH27	Chlorogenic acid	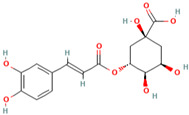	[19]
MH28	Neosperidin Dihydrochalcone	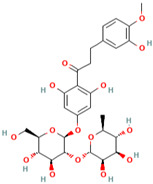	[19]
MH29	4-hydroxycinnamic acid	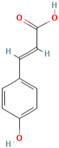	[19]
MH30	Taxifolin	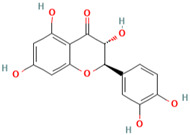	[19]
MH31	Rosmarinic acid	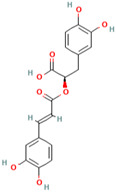	[19]
MH32	Myricetin	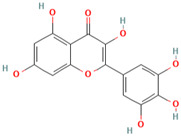	[19]
MH33	Baicalin	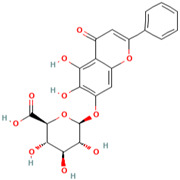	[19]
MH34	Melatonin	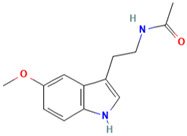	[20]
MH35	Trilobatin	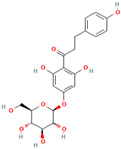	[21]
MH36	6″-O-coumaroyl-4′-O-glucopyranosylphloretin	NO	[21]
MH37	3‴-methoxy-6″-O-feruloy-4′-O-glucopyranosyl-phloretin	NO	[21]
MH38	Phloretin rutinoside	NO	[21]

NO represents that the chemical structure formula of the substance cannot be found accurately.

## Data Availability

No new data were created in the study.
